# *Serratia symbiotica* from the Aphid *Cinara cedri*: A Missing Link from Facultative to Obligate Insect Endosymbiont

**DOI:** 10.1371/journal.pgen.1002357

**Published:** 2011-11-10

**Authors:** Araceli Lamelas, María José Gosalbes, Alejandro Manzano-Marín, Juli Peretó, Andrés Moya, Amparo Latorre

**Affiliations:** 1 Institut Cavanilles de Biodiversitat i Biologia Evolutiva, Universitat de València, Valencia, Spain; 2 Unidad Mixta de Investigación en Genómica y Salud del Centro Superior de Investigación Salud Pública (Generalitat Valenciana) y del Institut Cavanilles de Biodiversitat i Biologia Evolutiva de la Universitat de València, València, Spain; Progentech, United States of America

## Abstract

The genome sequencing of *Buchnera aphidicola* BCc from the aphid *Cinara cedri*, which is the smallest known *Buchnera* genome, revealed that this bacterium had lost its symbiotic role, as it was not able to synthesize tryptophan and riboflavin. Moreover, the biosynthesis of tryptophan is shared with the endosymbiont *Serratia symbiotica* SCc, which coexists with *B. aphidicola* in this aphid. The whole-genome sequencing of *S. symbiotica* SCc reveals an endosymbiont in a stage of genome reduction that is closer to an obligate endosymbiont, such as *B. aphidicola* from *Acyrthosiphon pisum*, than to another *S. symbiotica*, which is a facultative endosymbiont in this aphid, and presents much less gene decay. The comparison between both *S. symbiotica* enables us to propose an evolutionary scenario of the transition from facultative to obligate endosymbiont. Metabolic inferences of *B. aphidicola* BCc and *S. symbiotica* SCc reveal that most of the functions carried out by *B. aphidicola* in *A. pisum* are now either conserved in *B. aphidicola* BCc or taken over by *S. symbiotica*. In addition, there are several cases of metabolic complementation giving functional stability to the whole consortium and evolutionary preservation of the actors involved.

## Author Summary

A critical issue in evolutionary biology is to find traits or organisms that provide evidence of the transition from one lifestyle to another, no matter how gradual the process may be. The evolutionary history of intracellular symbiosis, involving the transition of bacteria from free-living to obligate lifestyle, can reveal different types of bacteria, ranging from mere facultative to obligatory symbionts. Bacteria harboring traits of both types represent an important “missing link” in the history of this process, and that is precisely what we report here: the genome sequence and metabolic inferences of *S. symbiotica* from the aphid *C. cedri*, a bacterium that is closer to the obligate endosymbiont *B. aphidicola* from the aphid *A. pisum* than to the facultative *S. symbiotica* from the same aphid, whose genome has recently been sequenced. In addition, we provide metabolic evidence showing why *Buchnera* from *C. cedri*, which has lost some of the symbiotic capacities retained by other *Buchnera*, will not be replaced by *S. symbiotica*; but, conversely, both bacteria are evolving together towards the establishment of a powerful consortium with the aphid host.

## Introduction

Symbiotic associations are widespread in insects, being particularly well studied in aphids [1], [2]. They feed on phloem sap, which has unbalanced nitrogen/carbon content and is deficient in a number of nutrients that the aphids cannot synthesize and that are provided by *Buchnera aphidicola*, their primary endosymbiont [3], [4]. In addition to *B. aphidicola*, some aphid populations harbor additional facultative (or secondary symbionts) that are not required for growth or reproduction [5], which are sometimes transmitted horizontally [6]–[8]. Three main facultative symbionts have been found in aphids, i.e *Hamiltonella defensa*, *Regiella insecticola* and *Serratia symbiotica* [9]. Although their presence is not necessary for the maintenance of the aphid-*Buchnera* association, several studies have demonstrated that they can provide certain benefits to their hosts, such as influencing interactions with the host's natural enemies, or defense against environmental heat stress (revised in [5]). Most of the experimental studies on facultative symbionts in aphids have involved members of the subfamily Aphidinae, mainly in *Acyrthosiphon pisum*. In addition, the genome of the three above mentioned facultative symbionts from *A. pisum* have been sequenced [10]–[12]. These sequences have revealed that all three bacteria have lost the ability to synthesize most of the essential amino acids, although they retain active uptake mechanisms to import them. Thus, it seems that while *B. aphidicola* does not need the help of these facultative symbionts for host survival, they are dependent on *B. aphidicola* for amino acid provision when they infect *A. pisum*.

Furthermore, in the cedar aphid *Cinara cedri*, a member of the Lachninae subfamily, it was found that the co-existing endosymbiont *Serratia symbiotica* SCc was necessary for the survival of the *C. cedri* consortium. The genome of *B. aphidicola* BCc, the obligate endosymbiont of *C. cedri* with only 416 kb is the smallest *B. aphidicola* among all *Buchnera* genomes described, and one of the smallest bacterial genomes sequenced so far. Functional genome analysis revealed that with 362 genes, *B. aphidicola* BCc is able to support cellular life. However, its symbiotic role has been questioned because, contrary to other *Buchnera*, it was unable to fulfill some symbiotic functions [13]. Thus, it was postulated that the nutrients that *B. aphidicola* BCc cannot synthesize could be supplied by *S. symbiotica* SCc. Moreover, it was reported that this bacterium has characteristics of an obligate symbiont which differ from the other *S. symbiotica* described so far [7]. Microscopic analysis of *C. cedri* demonstrated that *S. symbiotica* SCc is confined in a second type of bacteriocytes whose presence is as abundant and homogeneous as *B. aphidicola* BCc [14]. In addition, both endosymbionts were found to be involved in tryptophan biosynthesis, supplying this essential amino acid to both their host and themselves [15]. Regarding the situation in the subfamily Lachninae, most members of the subfamily were found to have a massive presence of secondary symbionts, mainly *S. symbiotica* [7], [8]. Phylogenetic studies of these symbionts in all aphids from different subfamilies whose presence was reported, showed the existence of two clades, A and B, of *S. symbiotica* hypothetically playing two different roles: clade A is composed of facultative endosymbionts, whereas in clade B they would be obligate endosymbionts. Interestingly, *S. symbiotica* from *A. pisum* (herein S. *symbiotica* SAp) whose genome has recently been sequenced [12] belongs to clade A, whereas in this work we report a *S. symbiotica* genome belonging to clade B.

In the present study, we have carried out the genome sequencing and metabolic analysis of *S. symbiotica* SCc, the third partner of the *C. cedri* consortium. This bacterium has suffered an important genome size reduction to become a co-obligate symbiont. The comparative genomics of *S. symbiotica* SCc with *S. symbiotica* SAp and other obligate and facultative symbiotic bacteria, as well as with free-living *Serratia* relatives, mainly *S. proteamaculans* and the genetic and metabolic information retrieved from the genome sequence of *A. pisum* [16] and derived studies [17]–[19], provide an evolutionary scenario of how a symbiotic bacterial consortium is established.

## Results

### Genome of *S. symbiotica* SCc strain

General and specific features of the *S. symbiotica* SCc genome (CP002295) reflect its lifestyle as a host-restricted, mutualistic symbiont that invades host cells. The moderately reduced genome consists of a 1,762.765 bp circular chromosome with average G+C content of 29.22% (Table 1 and Figure S1). This chromosome size represents a 67.7% reduction compared to the free-living bacterium *S. proteamaculans* (CP000826) and a 36.8% reduction compared to *S. symbiotica* SAp (AENX00000000) [12]. A total of 711 putative genes have been described, with 672 protein coding genes (CDS), 36 tRNAs, 3 rRNAs and one tmRNA. It is worth mentioning that the ribosomal genes 23S and 5S are located on a chromosomal region separated from the 16S rDNA gene, a situation already detected in other obligate endosymbionts. However, *S. symbiotica* SAp, *H. defensa* HAp (CP001277.1) and *S. proteamaculans* have more than one copy of the ribosomal gene in an operon structure. Also, the number of genes coding for tRNA are closer to *B. aphidicola* BAp (NC_011833) than to facultative or free-living bacteria. Finally, 58 readily identifiable pseudogenes were present, which is a number closer to the one observed in primary than in secondary symbionts (Table S1).

**Table 1:** Comparison of *S. symbiotica* SCc genome features to those of obligate (*B. aphidicola* BCc and *B. aphidicola* BAp, from *C. cedri* and *A. pisum*, respectively) and facultative (*S. symbiotica* SAp, and *H. defensa* HAp, both from *A. pisum*) endosymbionts, and a free-living bacterial genome (*S. proteamaculans*).

	*B. aphidicola* BCc	*B. aphidicola* BAp	*S. symbiotica* SCc	*S. symbiotica* SAp	*H. defensa* HAp	*S. proteamaculans*
Genome size (bp)	424,849	652,115	1,762,765	2,789,218	2,169,363	5,495,657
Chromosome size (bp)	416,380	640,681	1,762,765	2,789,218	2,110,331	5,448,853
Plasmids	2	2	unknown	unknown	1	1
Plasmid size (bp)	8,849	11,434	-	-	59,032	46,804
Total number of genes	401	609	711	2,098	2,420	5,064
CDS (chromosome+plasmids)	357+7	562+9	672	2,098	2,094+54	4,891+51
rRNAs (16S, 5S, 23S)	1, 1, 1	1, 1, 1	1, 1, 1	5, 5, 5,	3, 3, 3	7, 8, 7
tRNAs	31	32	36	44	43	85
Pseudogenes	3	12	58	550	187+1	12
CDS average size (bp)	994.00	984.00	1,019,77	845	810.41	972.11
Coding density (%)	90.0	86.7	38.7	60.9	88.8	87.1
IGRs average size (bp)	135.80	126.90	1,672,01	204.27	240.26	165.67
G+C content	20.20	26.24	29.22	52.00	40.32	55.07
CDSs	21.40	28.00	38.51	52.53	40.99	56.34
1^st^+2^nd^ position	28.10	33.98	43.88	52.56	42.99	51.28
3^er^ position	7.77	14.23	28.46	52.48	36.99	66.47
IGRS (%)	9.30	16.10	26.53	45.43	37.26	45.71
Insertion Sequences (ISs)	No	No	No	Yes	Yes	Yes

The origin of replication was located between the genes *gidA* and *atpB*. The overall coding density is 38.7%, the lowest among insect endosymbionts described so far, including facultative symbionts of aphids, like *H. defensa* HAp (88.8%), *R. insecticola* RAp (ACYF00000000) (71.0%) or *S. symbiotica* SAp (60.9%) [10]–[12]. A very interesting feature of this genome is the average length of the intergenic regions (IGRs) (1,672 bp), which is much higher than in the other selected species (Table 1). A detailed analysis of these IGRs indicates that they do not show any traces of homology to coding regions from other bacteria. Due to the fact that protein-coding regions (CDSs) were found to be more G+C rich than non-coding regions [20], we decided to analyze the GC content distribution of *S. symbiotica* SCc and compare it with selected bacteria. We found a striking two-peak distribution of the genome GC content in *S. symbiotica* SCc, instead of the one peak found in any of the other selected organism [21] (Figure S2). To analyze where this two-peak distribution could originate, we took both *S. symbiotica* and plotted their CDSs and IGRs GC distribution separately. In *S. symbiotica* SCc, the IGRs mean GC content (27%) was found to fall very far from that of CDSs (38.74%), which contrasts with the case of *S. symbiotica* SAp where both IGRs and CDSs mean GC content only differed by 7% (Figure S3). In addition, the great number of pseudogenes in *S. symbiotica* SAp (550) also gave a similar GC mean of 51%. This points towards the last stages of genomic degradation of *S. symbiotica* SCc IGRs by displaying no evident homology with any known gene and displaying a high A+T content, a common feature arising in many bacterial endosymbionts in advanced stages of genome reduction. Finally, this genome has lost all the insertion sequences (IS) that are characteristic of free-living bacteria and facultative symbionts, as also observed in other bacterial genomes with long-term insect host associations [1], [2].

### Functional analysis of the predicted protein-coding genes

The protein genes of *S. symbiotica* SCc were classified according to COG categories [22] and compared with those of selected symbionts and free-living bacteria (Figure S4). The most relevant result is that *S. symbiotica* SCc has retained genes devoted to systems for which *B. aphidicola* BCc (NC_008513) was especially impaired compared with other *Buchnera*, such as biosynthesis of nucleotides, cofactors, lipid transport and metabolism, and cell envelope biogenesis. The only category in which it is clearly underrepresented is in amino acid metabolism (1.2% and 11.8% in *S. symbiotica* SCc and *B. aphidicola* BCc, respectively), which suggests the absolute metabolic dependence of *S. symbiotica* SCc on *B. aphidicola* BCc. Accordingly, *S. symbiotica* SCc possess many amino acid transport systems. Additionally, it has also preserved a wide range of transporters for other metabolites.

### Metabolic pathway reconstruction

*S. symbiotica* SCc has preserved all the steps of glycolysis as well as pentose phosphate pathway. Contrary to *S. symbiotica* SAp but similar to *Buchnera* spp, it has lost a functional TCA cycle, preserving only the genes *suc*C and *suc*D. These two genes may have been retained to produce succinyl CoA, necessary for lysine biosynthesis. As in other endosymbiont, acetyl-CoA could be used to produce acetate and ATP via the products of the genes *ack*A and *pta*, and conserve energy under oxygen-limiting conditions.

**Figure 1:**
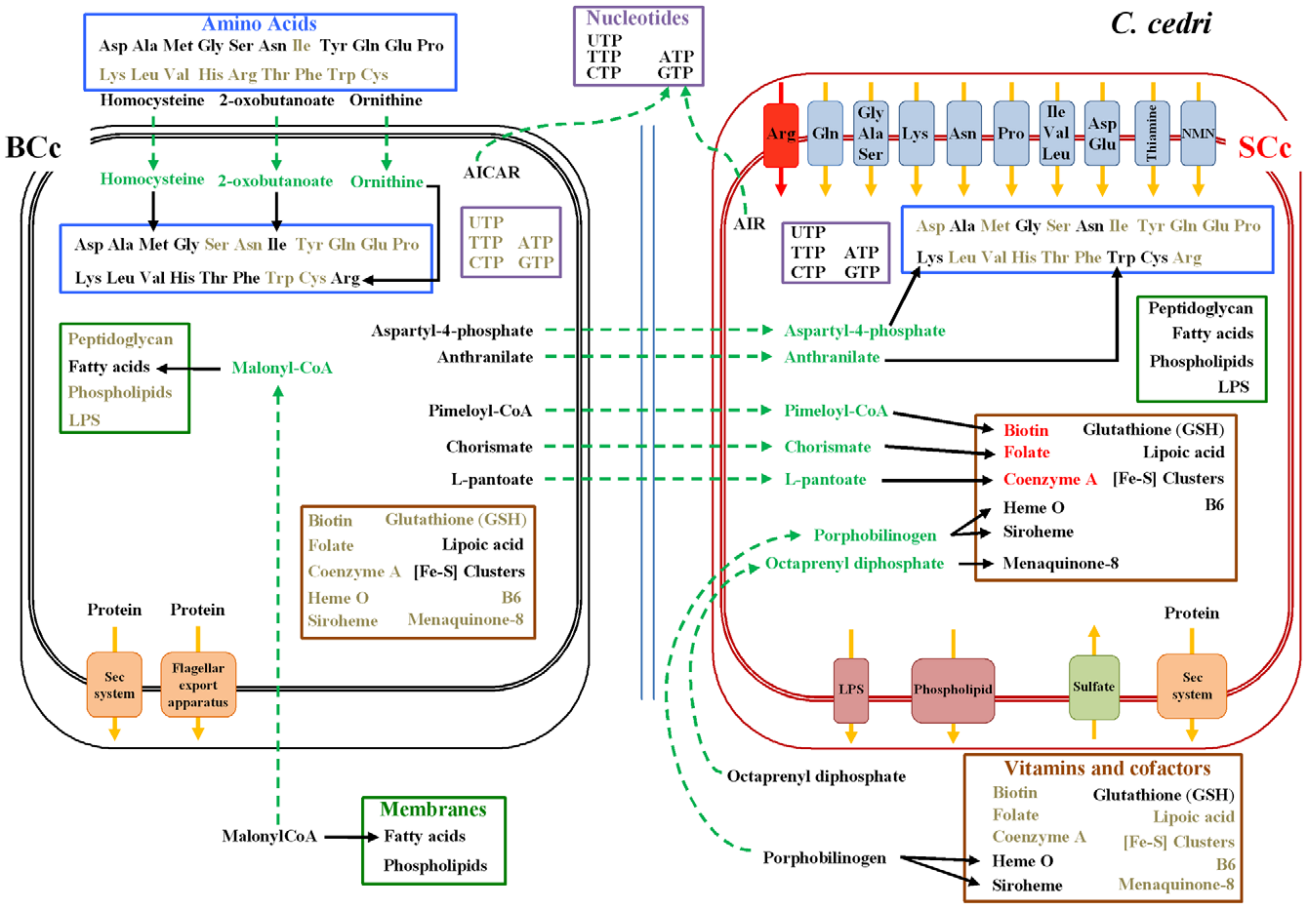
Inferred metabolism of amino acids, nucleotides, membrane compounds, cofactors, and vitamins of the *C. cedri*-consortium. *B. aphidicola* BCc is represented by three concentric black rectangles and *S. symbiotica* SCc by three red ones. The two internal rectangles represent internal and external bacterial membranes and the external rectangle represents the vesicle of the eukaryotic cell. The two blue lines represent the membrane in the bacteriocytes. Metabolites are black when synthesized, grey when not synthesized, red when their synthesis is not clear, and green for intermediates exchanged between partners. Black lines indicate intact pathways. Blue, pink and green squares on the membranes represent transporters (red indicating non-functional system). Orange boxes correspond to secretion systems.

**Figure 2:**
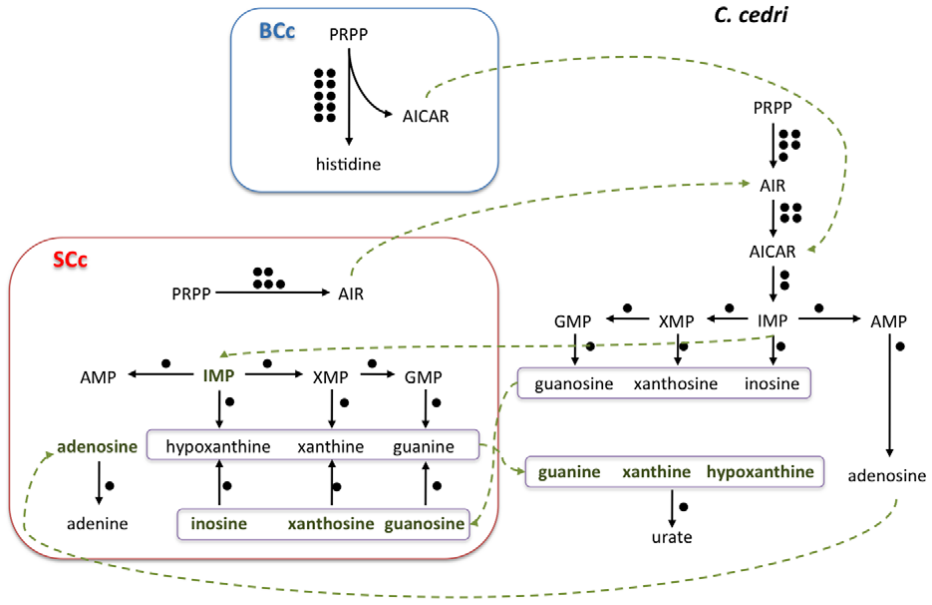
Outline of the putative synthesis of nucleotides by the consortium (*C. cedri, B. aphidicola* BCc, and *S. symbiotica* SCc). The number of genes involved in each pathway is shown as black circles beside them. The intermediate metabolites are colored in green. Green arrows indicate the movement of intermediary metabolites. In the case of aphid, the genes are postulated.

For most of the other pathways, one must postulate the involvement of one or even the two other members of the consortium, i.e. *B. aphidicola* BCc and the aphid (see Figure 1 for a summary of the shared metabolism). This is the case of the purine metabolism, where *S. symbiotica* SCc can only synthesize AIR from PRPP, but needs an external uptake of IMP to obtain AMP, GMP and XMP (Figure 2). Additionally, *S. symbiotica* SCc could salvage nitrogen bases from nucleotides or nucleosides that, when in excess, could in turn be transformed and eliminated as uric acid excretion by the aphid metabolism. This role is taken by *B. aphidicola* BAp in *A. pisum* [23]. On the other hand, *S. symbiotica* SCc possesses the complete machinery for pyrimidine biosynthesis. This is in clear contrast with the situation in *S. symbiotica* SAp where the purine *de novo* synthesis is complete, but to obtain pyrimidines it requires the nucleoside import, either from the aphid of from *B. aphidicola* BAp [12], [23].

**Figure 3:**
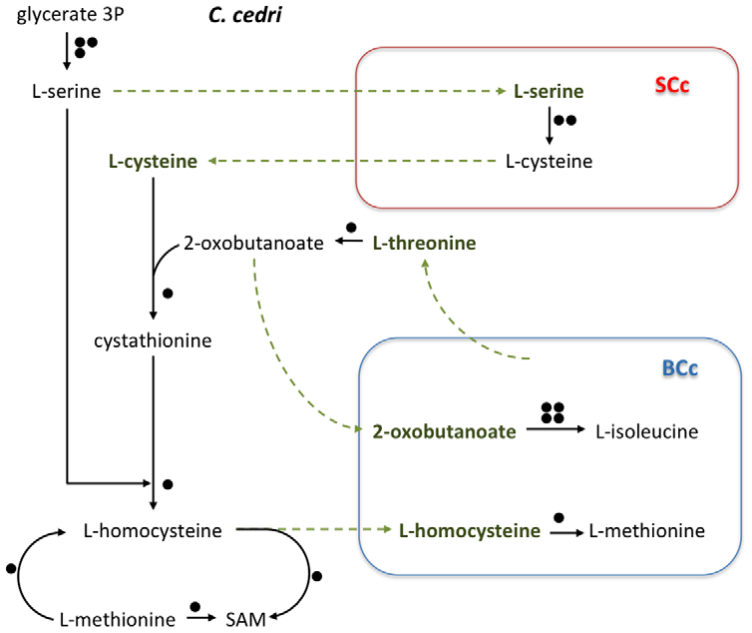
Outline of the putative synthesis of the amino acids serine, cysteine, isoleucine, and methionine by the consortium (*C. cedri*, *B. aphidicola* BCc, and *S. symbiotica* SCc). Rest as in Figure 2.

Most secondary endosymbionts retain the pathways for the synthesis of non-essential amino acids. However, *S. symbiotica* SCc has only preserved the pathways for alanine, cysteine and asparagine (Table 2). Regarding essential amino acid biosynthesis, *S. symbiotica* SCc has retained the ability to synthesize lysine and tryptophan provided that *Buchnera* metabolism supplies the respective precursors, i.e., aspartyl-4-phosphate and anthranilate (Figure 1). The latter situation (described elsewhere [15]) is similar in *S. symbiotica* SAp, which would also require exogenous anthranilate to synthesize tryptophan. However, in *A. pisum*, *B. aphidicola* BAp can provide the tryptophan as it possesses the complete pathway. A striking result relates the case of the non-essential amino acids serine and cysteine and the essential ones isoleucine and methionine, which, as shown in Figure 3, is necessary to postulate the metabolic complementation of all three members of the consortium to be synthesized. The aphid would produce serine from glicerate-3-p and then, *S. symbiotica* SCc could make cysteine. In turn, *B. aphidicola* BCc can provide threonine to the aphid to obtain the precursor of isoleucine. This is also similar in *A. pisum* and *B. aphidicola* BAp (17–19). Finally, *B. aphidicola* BCc could synthesize methionine, isoleucine and arginine with the external supply of homocysteine, 2-oxobutanoate and ornithine, respectively. We postulate that they come from the aphid, as might be the case in *B. aphidicola* BAp for methionine and isoleucine biosynthesis [17], [19].

**Table 2:** Metabolic capacity of amino acids biosynthesis by selected endosymbionts and free-living bacteria.

	*B. aphidicola* BAp	*B. aphidicola* BCc	*S. symbiotica* SCc	*S. symbiotica* SAp	*H. defensa* HAp	*R. insecticola* RAp	*S. proteamaculans*
Essential							
Histidine	+	+	−	−	−	−	+
Arginine	+	C-B	−	+*	−	−	+
Lysine	+	+	B-S	−	+	+	+
Threonine	+	+	−	−	+	+	+
Methionine	A-B	C-B	−	−	−	−	+
Valine	+*	+*	−	−	−	−	+
Isoleucine	A*-B	C*-B	−	−	−	−	+
Phenylalanine	+	+	−	+*	−	−	+
Tryptophan	+	B-S	B-S	−	−	−	+
Leucine	+*	+*	−	−	−	−	+
Non-essential							
Glycine	+	+	−	+	+	+	+
Proline	−	−	−	−	+	+	+
Glutamine	−	−	−	+	−	+	+
Cysteine	+	−	+	+	−	−	+
Asparagine	−	−	+	+	+	+	+
Alanine	+	+	+	+	+	+	+
Serine	+	+	−	+	+	+	+
Tyrosine	−	−	−	−	−	−	+
Glutamate	−	−	−	+	+	+	+
Aspartate	+	+	−	+	+	+	+

(+) synthesized, (−) not synthesized, (A-B) metabolic complementation between *A. pisum* and *B. aphidicola*, (C-B) postulated metabolic complementation between *C. cedri* an *B. aphidicola*, (B-S) metabolic complementation between *B. aphidicola* and *S. symbiotica*, (*) Absence of one enzyme involved in the pathway.

With regard to cofactors and vitamins, genome sequencing has revealed that *S. symbiotica* SCc is capable of synthesizing the same metabolites as *B. aphidicola* BAp as well as vitamin B6 (Table 3) although for biotin, folate and CoA, *S. symbiotica* SCc would require the provision of the respective precursors from *B. aphidicola* BCc, i.e. pimeloyl CoA, chorismate and L-pantoate (Figure 1). Clearly, *S. symbiotica* SCc has taken over these functions, which have been completely lost in *B. aphidicola* BCc. Moreover, *S. symbiotica* SCc could synthesize heme group in collaboration with the aphid, which must provide the porphobilinogen. This differs hugely from *S. symbiotica* SAp, which has preserved only four pathways (Table 3).

**Table 3:** Metabolic capacity of cofactor and vitamin synthesis by selected endosymbionts and free-living bacteria.

	*B. aphidicola* BAp	*B. aphidicola* BCc	*S. symbiotica* SCc	*S. symbiotica* SAp	*H. defensa* HAp	*R. insecticola* RAp	*S. proteamaculans*
Biotin	−	−	−	−	+	−	**+**
Lipoic acid	+	+	+	−	+	+	**+**
Folate	−	−	−	−	+	+	**+**
Glutathione	+	−	+	−	+	+	**+**
Heme	−	−	C-S	−	+	+	**+**
Siroheme	+	−	+	−	+	+	**+**
Nicotinate and nicotinamide	−	−	−	−	+	+	**+**
Pantothenate and CoA	−	−	−	+	−	+	**+**
Riboflavin	+	−	+	+	+	+	**+**
Thiamine	−	−	−	+	−	+	**+**
Ubiquinone	−	−	−	−	+	+	**+**
Menaquinone	−	−	−	−	+	+	**+**
Vitamin B6	−	−	+	+	+	+	**+**

(+) synthesized and (−) not synthesized. (C-S) postulated metabolic complementation between the aphid and *S. symbiotica*.

### Cell wall and membranes

**Figure 4:**
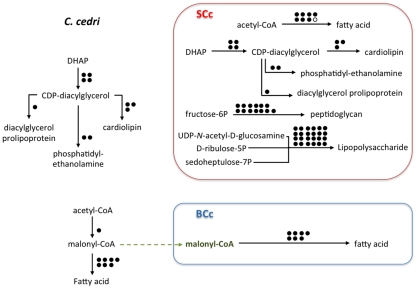
Outline of the putative synthesis of fatty acids, phospholipids (cardiolipin, phosphatidyl-ethanolamine, dyacilglycerol prolipoprotein), peptidoglycan, and lipopolysaccharide by the consortium (*C. cedri*, *B. aphidicoa* BCc, and *S. symbiotica* SCc). White circles representing absent genes. Rest as in Figure 2.

The genome sequencing of *S. symbiotica* SCc revealed that it retains the ability to synthesize peptidoglycan and liposaccharides to make its well-structured and complex membranes (Figure 4). This contrasts with its *B. aphidicola* partner, which has lost all the genes related to these functions [13]. Although *S. symbiotica* SCc retains the ability to synthesize these compounds, they are macromolecules and it is unlikely that they can enter *B. aphidicola* BCc. However, as can be seen in Figure S5, both bacteria maintain all three expected membranes, the two gram-negative and the external bacteriocyte-derived membrane.

### Pseudogenes, absent genes, and genome degradation

To gain insight into the pseudogenization process undergone by *S. symbiotica* SCc and *S. symbiotica* SAp in their respective lineages, we have compared the state of the annotated pseudogenes in both *Serratia* and the free-living *S. proteamaculans* (see Table S1 for details). From the 58 pseudogenes found in *S. symbiotica* SCc, two (*tuf* and *bam*A) have a duplicated functional copy. From the other 56, eighteen are also inactive genes (nine pseudogenes and nine absent genes) in *S. symbiotica* SAp, whereas 38 are active copies. Regarding the 311 chosen pseudogenes in *S. symbiotica* SAp (see Materials and Methods), as expected, most are absent in *S. symbiotica* SCc, and some are also absent in *S proteamaculans*, thus being strain specific A very interesting result is that sixteen of the *S. symbiotica* SAp pseudogenes are putatively active genes in *S. symbiotica* SCc (Table S1), thus indicating differential degradation fates in both *Serratia* lineages. Moreover, *S. symbiotica* SCc possesses 20 CDSs that are totally absent in *S. symbiotica* SAp.

### Synteny plots of *Serratia* species

**Figure 5:**
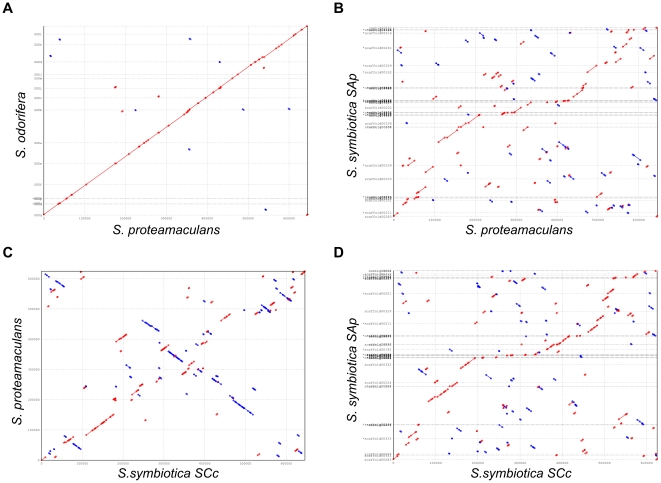
Synteny plots. Dot plots displaying syinteny between different species of *Serratia* in the shared groups of genes. (A) *S. proteamaculans* is taken as reference against *S. odorifera*. (B) *S. proteamaculans* is taken as reference against *S. symbiotica* SAp. (C) *S. symbiotica* SCc is taken as reference against *S. proteamaculans* and (D) *S. symbiotica SCc* is taken as reference against *S. symbiotica* SAp. Red dots, direct match; blue dots, reverse match.

In order to further compare the two intracellular *Serratia*, we performed the analysis of the synteny between both bacteria and also a comparison with free-living relatives. The results are shown in Figure 5 and clearly display the great number of rearrangements that occurred when the bacterium adopted an intracellular lifestyle, as is the case for both *S. symbiotica* compared to *S. proteamaculans*. The most interesting result is the comparison between *S. symbiotica* SCc and *S. symbiotica* SAp (panel D) where a series of rearrangements are found even in the biggest contigs, which suggest a past history of active mobile elements in *S. symbiotica* SCc, which are already unidentifiable in the current genome but still present in the *S. symbiotica* SAp.

## Discussion

Symbioses involving prokaryotes living in close relationships with insects have been widely studied from the genomic perspective [1], [2]. In the process towards host accommodation, symbionts experience a series of major genetic and phenotypic changes that can be detected by comparison with free-living relatives. Several scenarios could account for the evolution of symbiotic associations, from the first stages of free-living bacteria, through facultative symbiosis, to obligate symbionts. Of particular relevance is the association formed by the coexistence of several symbionts in a given host. Aphids are a good model to dissect the different stages of the integration process undertaken by the different symbionts coexisting therein. At present, the genome of *B. aphidicola* from five aphid species, belonging to different aphid lineages have been sequenced providing information of the last steps leading to obligate endosymbiosis [13], [24]–[27]. On the other hand, the genome of three facultative endosymbionts from the aphid *A. pisum* are also available [10]–[12]. They are in the early stages of transition from a free-living to symbiotic lifestyle, with *S. symbiotica* SAp probably representing the earliest stage of all three [12].

Our work indicates that *S. symbiotica* from *C. cedri* is a good candidate for a missing link between a facultative and an obligate insect endosymbionts. For comparative purposes, the two most relevant genomes are *B. aphidicola* BAp, the *Buchnera* with the biggest genome that does not need a second symbiont for aphid survival [17], [18], and *S. symbiotica* SAp because it is a *Serratia* symbiont, but in a much earlier step of the integration process [12].

Many features of the *S. symbiotica* SCc genome, such as the A+T content, the number of genes, the loss of *rec*A gene, as well as the total absence of ISs or other mobile DNA still present in all the facultative symbionts analyzed so far, are indicative of an obligate endosymbiont. It is worth mentioning that in *S. symbiotica* SAp, there are still a certain number of ISs, although because the genome sequence is incomplete, the exact number is not known. Moreover, transposases, plasmid-associated genes, and phage-associated genes can make up to 4% of the total number of genes [12]. On the other hand, *S. symbiotica* SCc has lost all the genes involved in bacterial pathogenesis that are still retained in *S. symbiotica* SAp. However, the *S. symbiotica* SCc genome size (1,763 kb) is intermediate between the two *A. pisum* symbionts, the obligate *B. aphidicola* (641 kb) and the facultative *S. symbiotica* SAp (*ca.* 2,789 kb), with non-coding DNA comprising a huge part of the genome. In fact, the coding density is extremely low (more than two times lower than that of *B. aphidicola* BCc), whereas the average size of the intergenic regions is extremely high (more than seven-fold that of *H. defensa*). According to our knowledge of prokaryotic genomes, these regions must correspond to ancient genes. However, in contrast with its related and recent symbiont *S. symbiotica* SAp, which has around 550 pseudogenes, in *S. symbiotica* SCc only 58 pseudogenes could be clearly identified [12]. These data support the postulated gradual process of genome degradation of the pseudogenes, ending up in their total disappearance in obligate bacterial endosymbionts [28]–[30]. In fact, if we substitute the size of the intergenic region in *S. symbiotica* SCc (1,672 bp on average) for the size of these regions in *B. aphidicola* BCc (135.8 bp on average), the chromosomal length would be 771,075 bp, a reduction of 43.7% and in the range of other obligate endosymbionts published so far (reviewed in [1], [2]).

The functional annotation of the *S. symbiotica* SCc genome indicated that its main symbiotic role would be the metabolism of cofactors, vitamins and nucleotides, whereas in *B. aphidicola* BCc it would be that of amino acid provider. However, the inferred metabolism of both endosymbionts has revealed a strong interdependence and a fine tuning of different biosynthetic pathways which, in some cases, probably also involves metabolic complementation with the aphid, as shown to occur in *A. pisum* [16]–[19], [23]. Overall, it seems that *B. aphidicola* BCc and *S. symbiotica* SCc in *C. cedri* jointly perform the metabolic functions that *B. aphidicola* BAp performs in *A. pisum*.

Another interesting feature relates to cell morphology. When *S. symbotica* SCc was first reported, its spherical morphology at the microscopic level was surprising [14], similar to the shape cells of *B. aphidicola* (Figure S5C), and different to the rod-shaped bacteria observed in *S. symbiotica* SAp [31] and in *S. symbiotica* from *C.tujafilina* [7]. These last two *Serratia* could be present in different locations in some individuals of the population, whereas *S. symbiotica* SCc are confined to their own bacteriocytes and occur in all individuals and at the same density as *B. aphidicola* BCc [14]. However, *S. symbiotica* SCc, like *S. symbiotica* SAp, has retained the genes involved in bacillary morphology (*mreB, mreC, mreD, mrdB*). These genes have been lost in all *B. aphidicola* genomes sequenced so far. At present, it is not clear whether these genes are being expressed or not, although the observed morphology is unexpected. The possible role played by the intracellular environment cannot be ruled out, possibly exerting some kind of effect on the morphology if those genes are expressed [31].

In summary, all the data presented (diversity in symbiont morphology, distribution and function) correlate with the existence of two different clades of *S. symbiotica* in aphids, at least, as also indicated by the phylogenetic analyses [7], [8]. The analysis of the synteny between *S. symbiotica* SCc and *S. symbiotica* SAp and the comparison with free-living *Serratia* indicate the great and different number of rearrangements undergone when the two bacteria adopted an intracellular lifestyle (Figure 5).

The comparison of the genome of all three secondary endosymbionts of *A. pisum*, *H. defensa*, *S. symbiotica* and *R. insecticola*, provides some clues to the scenario of how the *C. cedri* consortium came into being. These three bacteria, despite being facultative, could be retained by the aphid because they provide certain benefits to the host under particular conditions (for a review see [5]). Specifically, *S. symbiotica* SAp is involved in defense against environmental heat stress [32]–[34]. Due to the inactivation of some of their biosynthetic pathways, such as those related to the essential amino acid biosynthesis, over time, these bacteria have become dependent on the presence of *Buchnera*, and thus preserve active uptake mechanisms for their provision. On the other hand, as *B. aphidicola* is still undergoing a genome reduction process, some symbiotic functions may be lost and taken over by the second endosymbiont. When this happens, the consortium is established. The different agents involved in tryptophan biosynthesis in *A. pisum* and *C. cedri* is an amazing example of evolution towards the establishment of a consortium. In all the *B. aphidicola* strains, the two first genes of the tryptophan pathway, *trp*E and *trp*G, coding for anthranilate synthase, are either on a plasmid or in the chromosome, but always separated from the rest of the genes on the chromosome. Both *S. symbiotica* have lost these two genes, but preserve the other genes of the pathway (*trp*ABCD), implying *Buchnera* dependence for anthranilate provision. The main difference between both systems involves the obligate endosymbiont. In *A. pisum*, *Buchnera* can make tryptophan autonomously because it possesses the complete pathway, whereas in *C. cedri*, *Buchnera* has lost the *trp*ABCD genes, which are present in *Serratia*. This example could be enough to seal a consortium. Another case of metabolic collaboration between the two endosymbionts is the biosynthesis of lysine from aspartate. This pathway is complete in *B. aphidicola* BAp [19] whereas in *B. aphidicola* BCc only the first step, catalyzed by aspartokinase (*thr*A), takes place, whereas the other eight steps occur in *S. symbiotica* SCc (Figure 1). Moreover, additional cases of metabolic complementation might also exist during the synthesis of biotin, folate, and CoA in *S. symbiotica* SCc.

Finally, the fact that 36 active genes in *S. symbiotica* SCc are either pseudogenes, or absent genes in S. *symbiotica* SAp point towards different genome degradation processes in both *Serratia*. Such processes are context-dependent, i.e., the consequence of the different gene repertoire of the other agents when the association started, particularly the different genome composition of *B. aphidicola* in *A. pisum* or *C. cedri*.

In summary, here we report a missing link in the evolution from a facultative to an obligate endosymbiont. This is the case of *S. symbiotica* SCc when compared with *S. symbiotica* SAp, two different endosymbionts belonging to the same genus but in two different stages of the integration process leading to intracellular lifestyle: *S. symbiotica* SAp, a recently acquire facultative symbiont, and *S. symbiotica* SCc a recent co-obligate endosymbiont. We also gain insights into the establishment of a bacterial consortium between two co-obligate symbionts in aphids, *B. aphidicola* BAp and *S. symbiotica* SCc.

## Materials and Methods

### Aphid collection and total DNA extraction

*C. cedri* aphids were collected in Valencia, Spain. An enriched fraction of bacteriocytes was obtained as in [35], and then used to extract total DNA following a CTAB (Cetyltrimethylammonium bromide) method [36].

### Genome sequencing and assembling

The complete genome sequence of *S. symbiotica* SCc was obtained using single and paired-end shotgun reads from 454 pyrosequencing method (454 Life Science, Lifesequencing). The sequencing run generated 831,450 reads that assembled into 108,723 contigs using the GS De novo Assembler (version 1.1.03.24). Contigs expected to belong to the *Serratia* genome were identified by BLASTX searches against the GenBank non-redundant database [37], and reads associated with these contigs were extracted and reassembled to generate the *S. symbiotica* SCc genome. Reassembly produced 15 contigs. The order and orientation of some of the 15 contigs were predicted using the pair-ends information. All contig joins were confirmed using PCR amplification followed by Sanger sequencing. The tool Gap4.8b1 from Staden Package [38] was used for the total assembling of the Sanger sequences. This resulted in a single 1,762,765 bp contig with an average 454 (both single and paired-ends) coverage of 25.90×.

### Gene annotation and pseudogene prediction

The protein coding sequences (CDSs) were identified with the GLIMMER v3.02 program [39]. The ARTEMIS [40] program was used to check for the start and stop codons. Final annotation was performed using BLASTP comparison [37]. The tRNAscan [41] program was used to predict tRNAs, as well as other small RNAs, like tmRNA, the RNA component of the RNase P. Signal Recognition Particle RNA was identified by programs like SRPscan [42], as well as consulting the Rfam database [43]. Intergenic regions (IGRs) were manually analyzed by BLASTX and BLASTN to locate pseudogenes that were not found by GLIMMER. Then they were reanalyzed with Rfam, Pfam and NCBIs BLASTX [37], [43], [44] against the non-redundant database to look for any trace of coding fragments. Once the genome was finally annotated, the size of the genome, genes and intergenic regions was determined with ARTEMIS [40]. GC content was calculated by the on-line tool GeeCee (http://srs.nchc.org.tw/emboss-bin/emboss.pl?_action=input&_app=geecee).

### Genome GC difference and CDSs and IGRs GC content analysis

The nucleotide sequences from complete genomes, or from the contigs when the closed genome was unavailable, as occurred in *S. symbiotica* SAp and *R. insecticola*, were recovered from both *S. symbiotica* (SCc and SAp), S. proteamaculans (as a free-living *Serratia* representative), *H. defensa* and *R. insecticola* (as facultative aphid endosymbionts representatives), and *B. aphidicola* BCc (as a primary endosymbiont representative). Genomic GC difference was calculated as described in [21].

The IGRs and CDSs nucleotide FASTA files were extracted for both *S. symbiotica*, and GC content was calculated for each sequence.

### Inferred metabolism

The ORFs orthologous to known genes in other species were catalogued based on non-redundant classification schemes, such as COG (Clusters of Orthologous Groups of Proteins). The metabolic network was reconstructed using the automatic annotator server from KAAS-KEEG [45]. According to our genome annotation, each pathway was examined checking the BRENDA [46] and EcoCyc databases [47].

### Electron microscopy

*C. cedri* adult aphids were dissected (the head was removed) under a microscope in 0.9% NaCl fixed in 2% paraformaldehyde-2,5% glutaraldehyde in 0.2 M phosphate buffer (PB) for 24 h, and washed several times in 0.1 M PB. They were then post fixed in 2% osmium tetroxide in 0.1 M PB for 90 min in darkness, dehydrated in ethanol, and embedded in araldite (Durcupan, Fluka). Semithin sections (1.5 µm) were cut with a diamond knife, and stained with toluidine blue (Nikon Eclipse E800). Ultrathin (0.05 µm) sections were cut with a diamond knife, stained with lead citrate, and examined under transmission electron microscope (JEOLJEM1010).

### Analysis of presence/absence of pseudogens

Due to the fact that the *S. symbiotica* SAp genome is incompleted (12), CDSs that were present in more than one sequence (i.e. they had a gap spanning two contigs in a scaffold), as well as the pseudogenes without a name assigned or annotated as phage, transposase, integrase or hypothetical, were excluded from the analysis. This resulted in 311 pseudogenes and 2041 CDSs for this organism. First, the 58 pseudogenes present in *S. symbiotica* SCc were matched with their counterparts in *S. symbiotica* SAp and *S. proteamaculans* genomes (in both pseudogene and CDS databases for each organism). The CDSs were grouped by presence of same annotation or by BLAST (using pseudogenes as query and CDSs as subjects, with an e-value cut-off of 1e^−03^) and checked manually for function annotation. Also, BLASTX was run using pseudogenes as query against CDSs from all three *Serratia* and hits with a Bit Score Ratio > = 30 were selected and manually checked. Finally, genes not detected in *S. proteamaculans* (both against *S. symbiotica* SCc and *S. symbiotica* SAp CDSs and pseudogenes) were manually searched on the KEGG orthology database and in selected cases using BLAST with the pseudogene sequence against nr (restricted to *Serratia* taxonomy). The *S. symbiotica* SAp pseudogenes that did not match any *S. symbiotica* SCc pseudogenes in the analysis described above were matched to their CDS or pseudogene counterparts in both *S. symbiotica* (SCc and SAp) and *S. proteamaculans* in a similar fashion. COG categories from all pseudogenes, when not available, were obtained from both NCBI and KEGG. Plots were done using R [48].

### Synteny plots

Protein coding genes from *S. symbiotica* SAp, *S. odorifera* 4Rx13 (ADBX00000000) and *S. proteamaculans* 568 were downloaded from Genbank. *S. symbiotica* SAp CDSs that were in more than one sequence were omitted. We then used BLAST with an e-value cut-off of 1e-^05^ and 70% match cut-off. The results were clustered using MCL [49]. Common genes from all different *Serratia* species were extracted from each nucleotide FASTA file and ordered by contigs (when not in a single one), and Promer from the Mummer package [50] was used to plot the comparisons. *S. odorifera* 4Rx13 was used to exemplify the contig rearrangement algorithm due to the low number of contigs present in the genome annotation.

## Supporting Information

Figure S1 Circular map of S. *symbiotica* SCc genome. From outer to inner circles: COG categories in both strands, tRNAs (grey), rRNAs (green), GC skew (red: positive skew, blue: negative skew), G+C content ( purple and orange, % value above and below average, respectively).
(TIF)

Figure S2 Distributions of GC differences in selected bacteria. The histograms show the distribution for the GC difference (see Materials and methods). The blue curves are epirical density estimates.
(TIF)

Figure S3 CDSs and IGRs GC content distributions in *S. symbiotica* SCc (A, B) and *S. symbiotica* SAp (C,D), respectively. The blue curves are empirical density estimates, whereas the red vertical lines represent the sample mean.
(TIF)

Figure S4 COG distribution of protein-coding genes in the *S. symbiotica* SCc compared with some obligate and some free living bacterial distributions.
(TIF)

Figure S5 Electron micrograph of the cell (A) *B. aphidicola* BCc, (B) *S. symbiotica* SCc and (C) bacteriocytes of *B. aphidicola* and *S. symbiotica*. mit: mitochondria, m_i_: inner membrane, m_o_: outer membrane, m_v_: eukaryotic vesicle membrane, n: nucleus of bacteriocyte.
(TIF)

Table S1 Pseudogene state comparison of *S. symbiotica* SAp, *S. symbiotica* SCc and *S. proteamaculans* (Spro) and *S. symbiotica* SAp missing genes.
(DOC)
