# Supplementary figures and images for: Serratia symbiotica from the Aphid Cinara cedri: A Missing Link from Facultative to Obligate Insect Endosymbiont
Source: PLoS Genet. 2011 Nov 10;7(11):e1002357. doi: 10.1371/journal.pgen.1002357 (PMC3213167; doi:10.1371/journal.pgen.1002357)

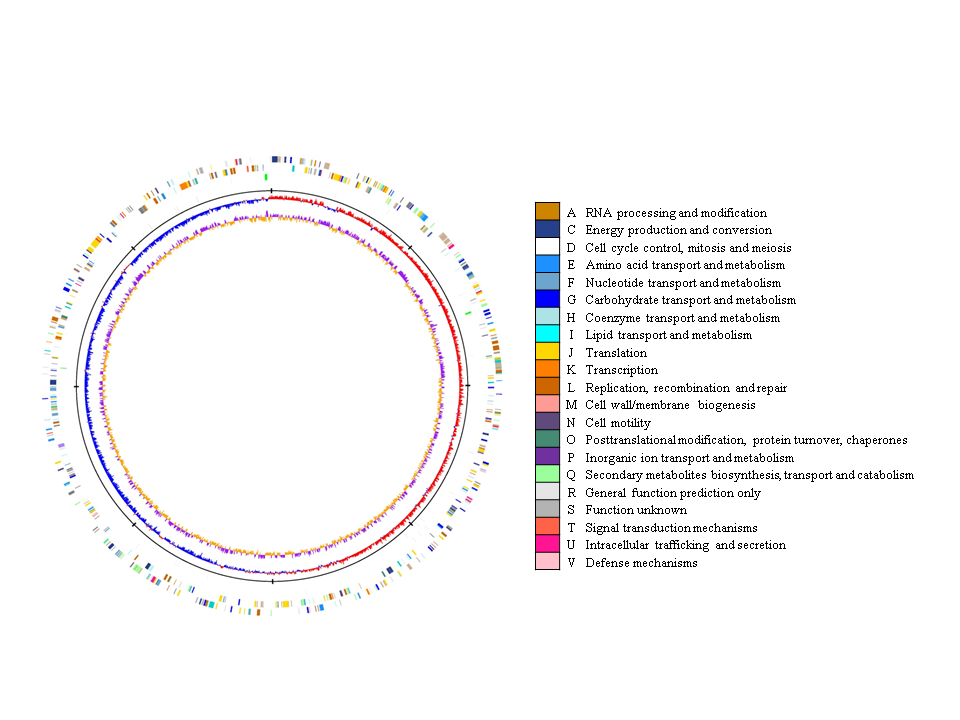

Supplement: Figure S1 — Circular map of S. symbiotica SCc genome. From outer to inner circles: COG categories in both strands, tRNAs (grey), rRNAs (green), GC skew (red: positive skew, blue: negative skew), G+C content ( purple and orange, % value above and below average, respectively). (TIF) [file pgen.1002357.s001.tif]

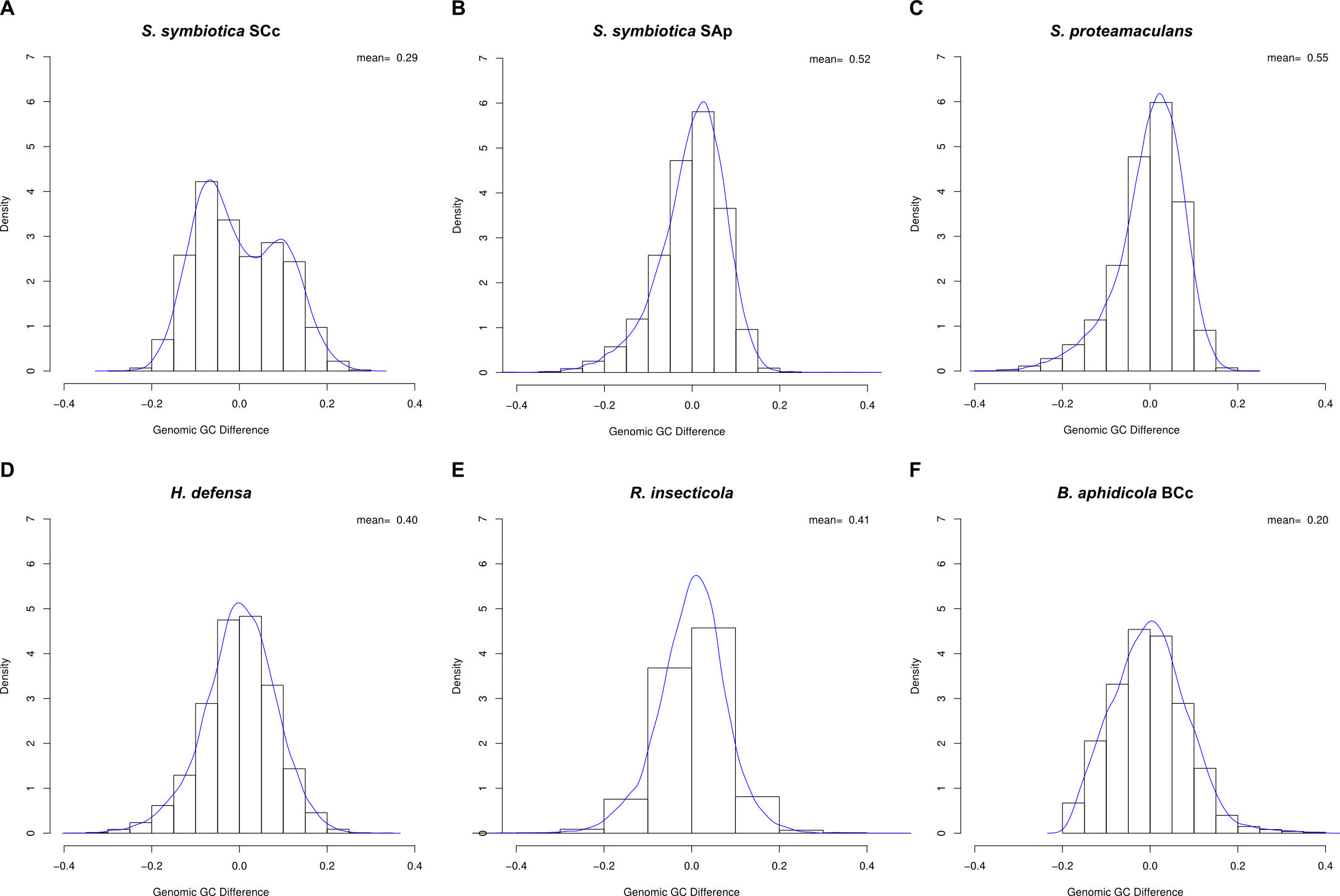

Supplement: Figure S2 — Distributions of GC differences in selected bacteria. The histograms show the distribution for the GC difference (see Materials and methods). The blue curves are epirical density estimates. (TIF) [file pgen.1002357.s002.tif]

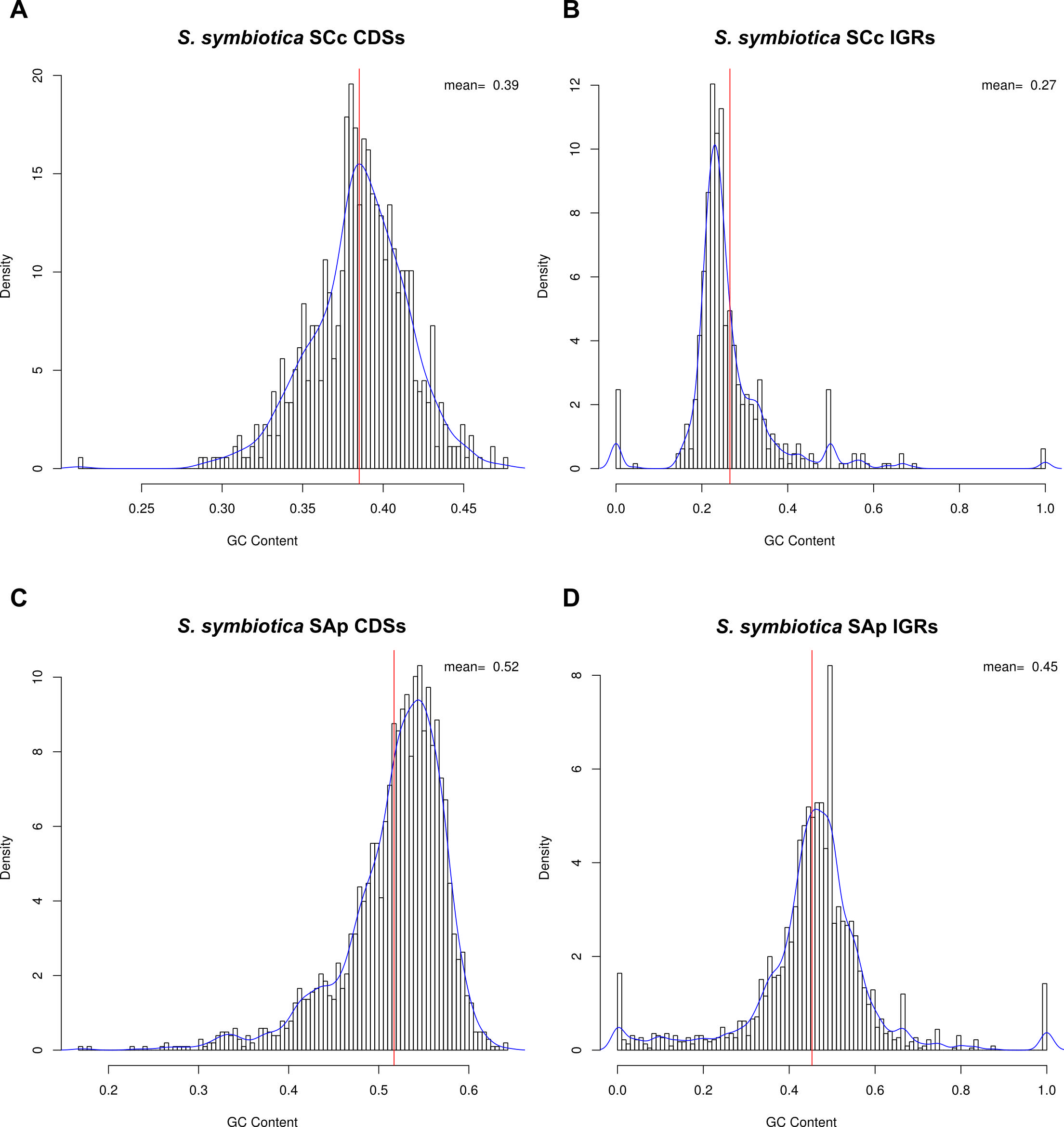

Supplement: Figure S3 — CDSs and IGRs GC content distributions in S. symbiotica SCc (A, B) and S. symbiotica SAp (C,D), respectively. The blue curves are empirical density estimates, whereas the red vertical lines represent the sample mean. (TIF) [file pgen.1002357.s003.tif]

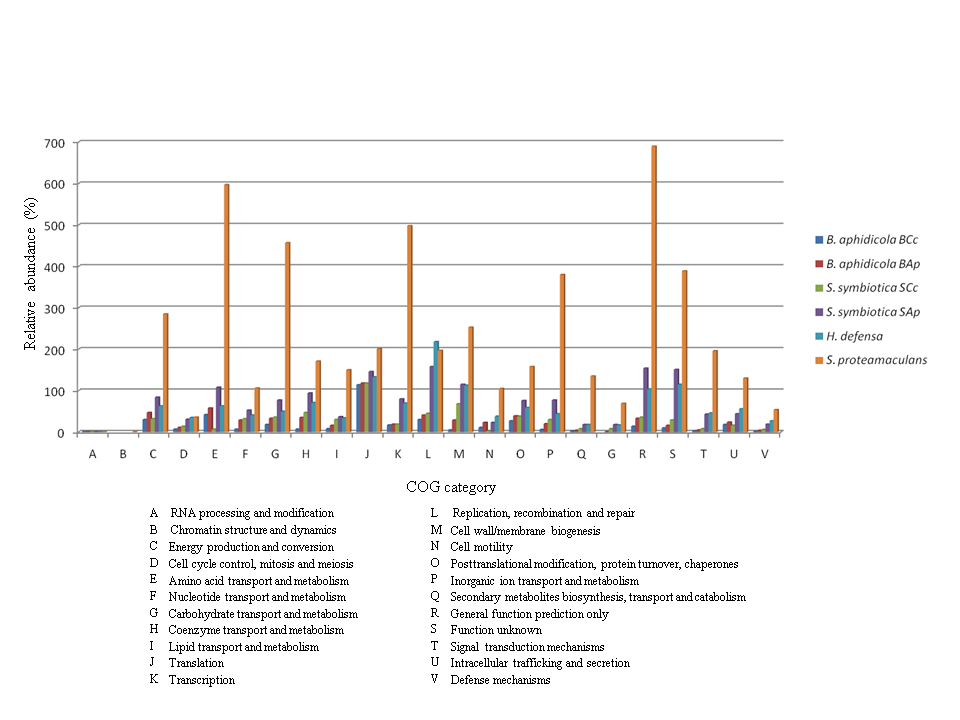

Supplement: Figure S4 — COG distribution of protein-coding genes in the S. symbiotica SCc compared with some obligate and some free living bacterial distributions. (TIF) [file pgen.1002357.s004.tif]

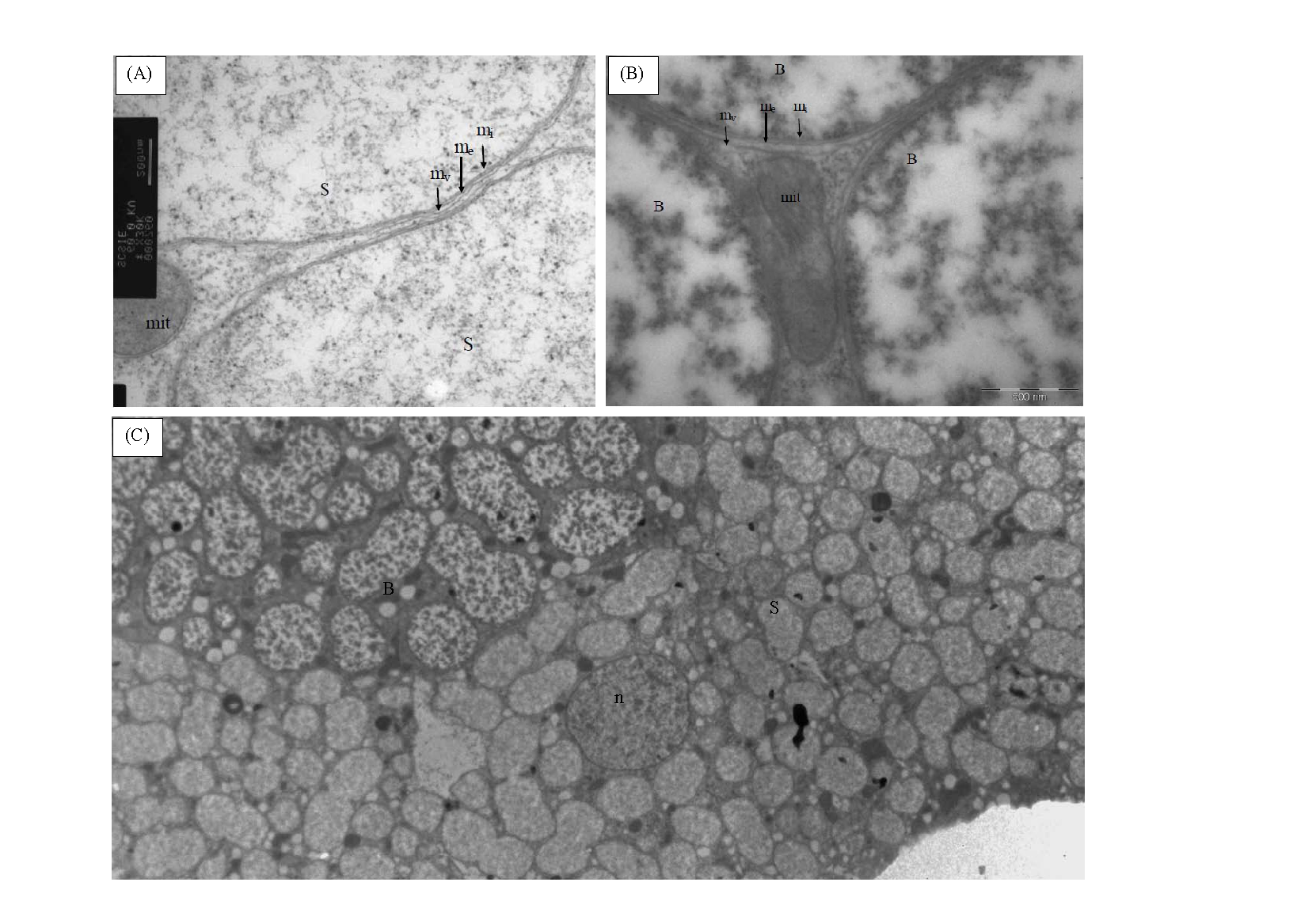

Supplement: Figure S5 — Electron micrograph of the cell (A) B. aphidicola BCc, (B) S. symbiotica SCc and (C) bacteriocytes of B. aphidicola and S. symbiotica. mit: mitochondria, mi: inner membrane, mo: outer membrane, mv: eukaryotic vesicle membrane, n: nucleus of bacteriocyte. (TIF) [file pgen.1002357.s005.tif]
