# Supplementary material for: Serratia symbiotica from the Aphid Cinara cedri: A Missing Link from Facultative to Obligate Insect Endosymbiont
Source: PLoS Genet. 2011 Nov 10;7(11):e1002357. doi: 10.1371/journal.pgen.1002357 (PMC3213167; doi:10.1371/journal.pgen.1002357)
Supplement: Table S1 — Pseudogene state comparison of S. symbiotica SAp, S. symbiotica SCc and S. proteamaculans (Spro) and S. symbiotica SAp missing genes. (DOC) [file pgen.1002357.s006.doc]

**Table S1. Analysis of the presence/absence of pseudogenes in *S. symbiotica* SCc; *S. symbiotica* SAp and *S. proteamaculans* (Spro).**

| **COG** | **Gene(s)** | **SCc** | **SAp** | **Spro** |
| --- | --- | --- | --- | --- |
| C | *dctA* | + | ψ/+ | + |
|  | *atpI, citF, cydA, cydC, fldB, frdA, glpK, ldhA, leuB, mchF, nqrA, nqrB, nqrF, nuoC, nuoF, nuoG, nuoJ, nuoL, nuoM, nuoN, putA, ydiJ, ydjA, yfhL* | - | ψ | + |
|  | *yqcA* | - | ψ/+ | + |
|  | *ppc* | ψ | + | + |
| D | *ttcA* | - | ψ | * |
|  | *ftsN, zipA* | ψ | + | + |
| E | *cysD, ansP, glnQ, glnP, glnH* | + | - | + |
|  | *argD, astE, avtA, cysM, gcvT, ggt, gsiB, gsiC, hisC, hisD, hisG, hisH, hisI, ilvA, ilvE, leuA, metB, metC, oppA, oppB, pabB, pabC, ptrB, sdaA, selA, selD, speA, trpE* | - | ψ | + |
|  | *ilvB, metA, oppF, proX, yifK,trpG* | - | ψ/+ | + |
|  | *aroQ, aspC, pheA, thrB, yfbQ* | ψ | + | + |
|  | *artI, artM, artP, artQ, metE* | ψ | - | + |
|  | *thrA, thrC* | ψ | ψ | + |
|  | *yeeF* | ψ | ψ/+ | + |
| F | *pyrC* | + | - | + |
|  | *gpp, guaC, nrdD, nrdE, nrdI, upp, xanP* | - | Ψ | + |
| G | *fruA, fruB, fruK, pykA* | + | Ψ | + |
|  | *arnD, bcr, bglF, entS, glk, rbsC, rbsD, treB, yadE, ydhC* | - | Ψ | + |
|  | *mtlA* | - | Ψ+ | + |
|  | *cpsG* | Ψ | Ψ | + |
| H | *ptpS, cysG* | + | - | + |
|  | *bioA, bioB, hemC, hemD, pdxY, pnuC* | + | Ψ | + |
|  | *bioF, entC, hemL, menA, panC, panE, rraA* | - | Ψ | + |
|  | *dfp, hemX, ygdL* | - | Ψ | + |
| I | *acs, fadL, folM, menE, pgpB* | - | Ψ | + |
| J | *queA, rsmB* | + | Ψ | + |
|  | *dusC, rnd, rsmF, trmA, yjgF, yoaB* | - | Ψ | + |
|  | *arnA* | Ψ | Ψ | + |
|  | *tuf* | ψ/+ | + | + |
| K | *asnC, cobB, cueR, dpiA, fliA, gcvA, hdfR, hepA, iscR, lrhA, rob, slyA, treR, ybaO, yoaA* | - | Ψ | + |
|  | *ybtA* | - | Ψ | - |
|  | *dgsA, rbsR* | - | ψ/+ | + |
|  | *fabR, rof* | Ψ | + | + |
| L | *priB, uvrC, uvrA* | + | - | + |
|  | *nudF* | + | Ψ | + |
|  | *gyrA* | + | ψ/+ | + |
|  | *helD, hrpA, hrpB, polB, rarA, sbcC, sbcD, umuC, xthA, ybaV, yrdD* | - | Ψ | + |
|  | *recA* | - | ψ/+ | + |
|  | *rhlE* | Ψ | + | + |
|  | *nudE* | Ψ | + | - |
|  | *seqA* | Ψ | Ψ | + |
|  | *xerC* | Ψ | ψ/+ | + |
| M | *mltE, lpp* | + | - | + |
|  | *mrcA, rseP, thiK* | + | Ψ | + |
|  | *lpxK* | + | ψ/+ | + |
|  | *amiC, arnB, cpsB, gutQ, mchE, nlpE, rlpA, slt, tsx* | - | Ψ | + |
|  | *waaL* | - | Ψ | - |
|  | *mepA, mscL* | - | ψ/+ | + |
|  | *bamC, rfaQ, rfbB, rffD, rffG, rffH, rffM, wzzE* | Ψ | + | + |
|  | *wbpZ* | Ψ | + | - |
|  | *gmd* | ψ | - | - |
|  | *rfe, rffE* | Ψ | Ψ | + |
|  | *bamA* | Ψ/+ | + | + |
| **N** | *hofC, mrfC, pilL, ybgD, ybgQ* | - | Ψ | + |
|  | *mrfD, pilT* | - | ψ/+ | + |
|  | *pilR, pilV, triJ* | - | ψ/+ | - |
| O | *cysU* | + | Ψ | + |
|  | *clpA, degS, fklB, glnD, glnE, trxC, yacC, ybbJ, ybbN, yifB* | - | Ψ | + |
|  | *grxC, pcm, sspA* | Ψ | + | + |
| P | *apaG, cysN, cysC, ygdQ* | + | - | + |
|  | *cysJ, thiP* | + | Ψ | + |
|  | *cysA* | + | ψ/+ | + |
|  | *copA, fecA, fecB, fepB, fepD, fes, fhuA, fieF, kefB, mgtA, modA, modB, nhaB, opgG, pstA, pstS, tauB, ybaL, yfjD, yjbB* | - | Ψ | + |
|  | *katE, yrbG* | - | ψ/+ | + |
|  | *cysP* | Ψ | + | + |
| Q | *entF, ftsP, tauD, ycgM* | - | Ψ | + |
|  | *rlmA* | - | ψ/+ | + |
| R | *sufE* | + | - | + |
|  | *yceG* | + | Ψ | + |
|  | *csdE, gsiA, menH, recX, rlmI, sgrR, torD, yafJ, ybbO, ybhB, ycaI, yciV, yfcA, yhjJ, yjjK, ypfJ* | - | Ψ | + |
|  | *queC, tmcA, ydjN, yheS* | - | ψ/+ | + |
|  | *rsgA, yggS* | Ψ | + | + |
|  | *ubiB* | Ψ | Ψ | + |
| S | *yeiH, yhdP* | + | Ψ | + |
|  | *dedA, rsmE, ybaP, ycaO, ycfD, yeaC, yfiH, yggE, yheO, yjjX* | - | Ψ | + |
|  | *dedD, yibQ, yigA, zapA* | Ψ | + | + |
|  | *mtfA, ybiS* | Ψ | - | + |
|  | *smg* | Ψ | Ψ | + |
| T | *apaH* | + | - | + |
|  | *arcB, cpxA, csrD, proQ, qseF, rcsD, rseB, rstB, traR, yfeA, yjiY* | - | Ψ | + |
|  | *rcsC* | - | ψ/+ | + |
|  | *envZ, spoT* | Ψ | + | + |
| U | *shlB* | - | Ψ | + |
|  | *trbC* | - | Ψ | - |
|  | *triC, triH* | - | ψ/+ | - |
| V | *mdtK* | + | Ψ | + |
|  | *ampD, ampE, dinF, mdtB, ybtP* | - | Ψ | + |
|  | *mdtC* | - | ψ/+ | + |
| None | *ybjN* | + | - | + |
|  | *yceB* | + | Ψ | + |
|  | *barA, srfC, syd, yfcD, yjaH* | - | Ψ | + |
|  | *irp1, irp2, traY, yspA, yspC* | - | Ψ | - |
|  | *rffT* | - | ψ/+ | + |
|  | *traC* | - | ψ/+ | - |
|  | *bssS* | Ψ | - | + |
